# Immersive and interactive visualization of 3D spatio-temporal data using a space time hypercube: Application to cell division and morphogenesis analysis

**DOI:** 10.3389/fbinf.2023.998991

**Published:** 2023-03-08

**Authors:** Gwendal Fouché, Ferran Argelaguet, Emmanuel Faure, Charles Kervrann

**Affiliations:** ^1^ Inria de l’Université de Rennes, IRISA, CNRS, Rennes, France; ^2^ LIRMM, Université Montpellier, CNRS, Montpellier, France; ^3^ Inria de l’Université de Rennes, Rennes, France; ^4^ UMR144 CNRS Institut Curie, PSL Research University, Sorbonne Universités, Paris, France

**Keywords:** spatio-temporal visualization, dimension reduction, space-time cube, interaction, virtual reality, immersive analytics

## Abstract

The analysis of multidimensional time-varying datasets faces challenges, notably regarding the representation of the data and the visualization of temporal variations. We propose an extension of the well-known Space-Time Cube (STC) visualization technique in order to visualize time-varying 3D spatial data, taking advantage of the interaction capabilities of Virtual Reality (VR). First, we propose the Space-Time Hypercube (STH) as an abstraction for 3D temporal data, extended from the STC concept. Second, through the example of embryo development imaging dataset, we detail the construction and visualization of a STC based on a user-driven projection of the spatial and temporal information. This projection yields a 3D STC visualization, which can also encode additional numerical and categorical data. Additionally, we propose a set of tools allowing the user to filter and manipulate the 3D STC which benefits the visualization, exploration and interaction possibilities offered by VR. Finally, we evaluated the proposed visualization method in the context of 3D temporal cell imaging data analysis, through a user study (n = 5) reporting the feedback from five biologists. These domain experts also accompanied the application design as consultants, providing insights on how the STC visualization could be used for the exploration of complex 3D temporal morphogenesis data.

## 1 Introduction

The analysis of time-varying 3D spatial data faces a number of challenges due to the complexity of the captured 3D structures, which require the need of adapted visualization methods. Immersive analytics Chandler et al. (2015), as reported by Skarbez et al. (2019), leverages large field of view displays, stereoscopic rendering, motion parallax and rich spatial 3D interactions in order to increase spatial understanding, decrease information clutter and help with visual analytics tasks. In particular, the use of virtual reality (VR) can help with flexible exploration and interaction with complex time-varying 3D spatial data. For example, according to Laha et al. (2012), the enhanced depth perception offered by stereoscopy improves the visualization of details in volumetric data, while having a large workspace allows for the juxtaposition of multiple coordinated and interactive views (Johnson et al., 2019).

**FIGURE 1:**
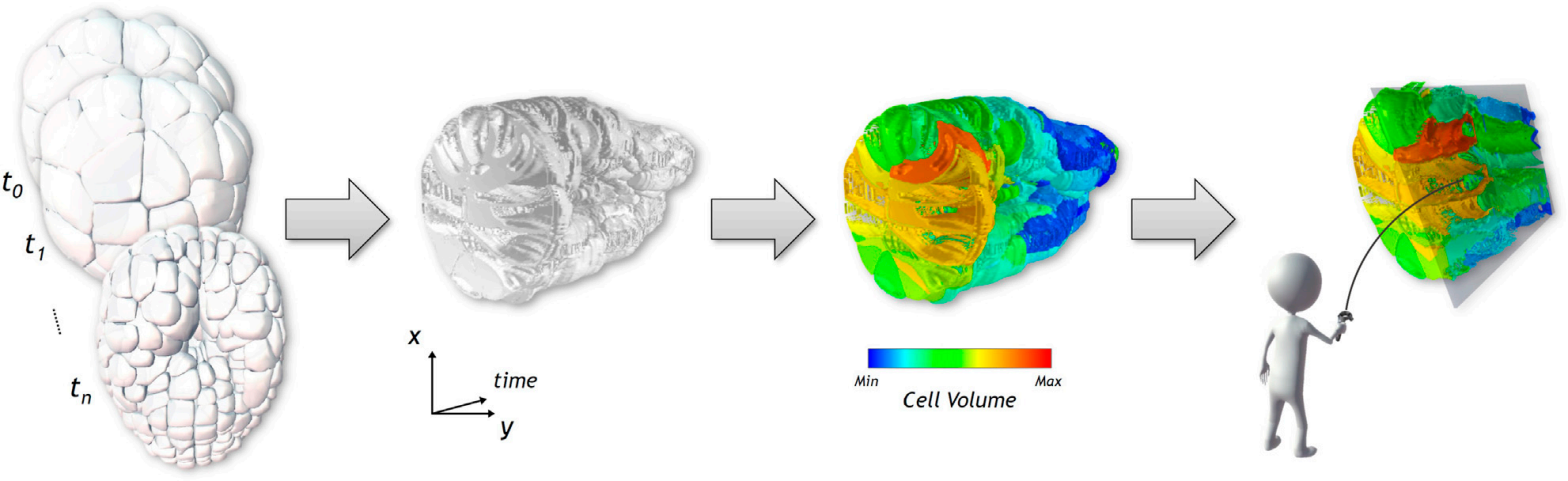
From a cross-section on the 3D surface-based temporal data shown on the first figure, we generate a Space-Time Cube visualization, displayed in the second image, showing the evolution over time of the spatial data of the cross-section displayed on the *x* and *y* axes. The third picture shows how the visualization can be enriched with numerical and categorical data using different color coding. A set of interaction tools help the user to explore the generated visualization, as seen in the last image.

Methods from the field of Immersive Analytics could notably be beneficial for biological imaging, where large time-varying datasets are more and more present (Long et al., 2012), and constraints emerge from the growing spatio-temporal resolutions and the complexity of the obtained images. For instance, Lattice Light-Sheet Microscopy (Chen et al., 2014) allows the capture of volumes at a resolution of a few hundred nanometers at a few seconds interval. This technology is notably used for recording embryo development (Guignard et al., 2017), creating terabytes of raw data, in contexts of analysis in embryology, morphodynamics, and intracellular dynamics. In such context, extracting surface meshes to describe volumes (see Figure 1, left) is a commonly used solution in order to display and analyze data, as proposes the web-based application MorphoNet, by Leggio et al. (2019). In such complex datasets, identifying the relations between objects, categorical data and their joined evolution over time, becomes a challenge for end users. While, software widely used by biology experts, such as ImageJ (Rasband, 1997) or Imaris (Oxford Instruments plc., 1993), can handle the viewing of such data, they lack adequate tools to identify and visualize this type of relations.

The main objective of this paper is to propose visualization methods enabling the comparison of 3D spatio-temporal data leveraging the benefits of immersive analytics. For this purpose, we propose an extension of the Space-Time Cube (STC) visualization technique (see Bach et al., 2014) to 4D spatio-temporal data, named hereinafter Space-Time Hypercube (STH). Conceptually, the STH considers that the data to visualize lays in a 4D hypercube with three spatial dimensions and one temporal dimension. To enable the direct visualization of the 4D hypercube, we extended the classic operations applicable to STCs to generate various meaningful 3D visualizations, that can be juxtaposed to obtain a compact overview of spatio-temporal data. Using such extended operations, we propose a projection method, independent of the type of spatial representation (e.g., mesh, volumetric), which projects the hypercube into a 3D volume that can be directly visualized. The projection operation relies on a user-defined cross-section on the spatial dimension. This cross-section is computed along the temporal dimension and stacked together into a 3D volume, hence a STC, which can be enriched with numerical and categorical data (see Figure 1). The enhanced depth perception of VR is expected to improve the visual extraction of meaningful structures of the generated STC and the use of 3D interaction (LaViola Jr et al., 2017) is expected to ease the exploration and interaction with the STC, as suggested in several works, in general cases (Loomis and Knapp, 2003) and specifically for STCs (Bach et al., 2017a; Ssin et al., 2019; Filho et al., 2019). Furthermore, the enlarged interaction space of VR allows the juxtaposition of the STC and snapshots of the original spatio-temporal data, giving additional context required to analyse complex multidimensional data. This juxtaposition enables the synchronized exploration and manipulation of the two visualizations. In order to provide adequate tools for basic analysis of our use case data, we considered the input from domain experts to improve the design of the application. Finally, the paper presents a use-case illustrating the usages of the STH to generate meaningful visualization of spatio-temporal data in embryo developmental studies, as well as an evaluation with domain experts.

In summary, the main contributions of the paper are:

• An extension of the well-known Space-Time Cube technique as an abstraction of 3D temporal data, named Space-Time Hypercube;
• A projection operator of the 4D STH to generate a 3D STC that can be visualized in an Immersive Analytics application;
• An evaluation of the proposed method on embryology use cases by domain experts.

## 2 Related work

Time-varying data are more and more present in scientific visualization, and numerous methods have been developed to visualize the time component and space-time relationships. Such methods can focus on the visualization of point clouds (Li et al., 2013; Lu and Shen, 2008), trajectory datasets with few (Amirkhanov et al., 2019; Andrienko et al., 2017) or large amounts of instances (Neuroth et al., 2019; Huang et al., 2015; Liu et al., 2016; Hurter et al., 2009), simulated flow data (Waser et al., 2010; Schindler et al., 2012) and surface or volumetric datasets (Kim et al., 2017). Immersive visualization methods are also becoming commonplace (Hurter et al., 2018; Homps et al., 2020). However, due to the vast literature in this domain, we constrain the state of the art to visualization methods adapted to datasets representing time-varying 3D spatial data with a particular focus on Space-Time Cube visualizations methods, which is the main scope of the paper. For a comprehensive review of spatio-temporal visualization methods, we refer the reader to the following comprehensive works: (Aigner et al., 2011; Andrienko and Andrienko, 2013; Bai et al., 2020).

### 2.1 Visualization of time-varying volume data

A number of approaches exist to visualize time-varying data: dynamic methods, implying animation and interaction, and static visualizations of either the full data, or extracted lower-dimension data.

#### 2.1.1 Dynamic visualizations

Animation is an intuitive method to explore time evolution in 3D temporal datasets. In designs using animation, time increases automatically, showing the evolution of the data through time. The work of Coffey et al. (2012) showed that animated and interactive design choices for time exploration can decrease the amount of error in analysis tasks. They also suggested that hybrid methods that would implement interactive, animated and/or static time exploration designs could compensate for the flaws of other methods. For instance, Akiba et al. (2010) proposed Aniviz, an interactive animation interface enabling the exploration of time-varying volumetric data by editing a time-dependent transfer function. The user can specify display, animation and data related parameters. Another example is FluoRender Wan et al. (2012), which enabled the visualization of 4D datasets captured using confocal microscopy using a playback mechanism with 4D tracking capabilities. However, animation can be less adequate for comparison tasks (Kim et al., 2017) or the analysis of space-time-value relationships (Woodring and Shen, 2006). There is a higher cognitive load for the user who has to remember the state of the data between different moments, which can limit the observation of details, as the slogan “*Eyes Beat Memory*” suggests. Solteszova et al. (2020) proposed to use time-warping in animations to cope with this issue. Their method allows the user to manipulate time evolution of a video or a selected point of interest in the video. Yet, other solutions for the issue suggested by the slogan can be found in static visualizations.

#### 2.1.2 Static visualizations

Woodring and Shen (2006) proposed a solution for static temporal visualization for time-varying volumetric data, based on set and numerical operations to filter the displayed information, reduce occlusion and focus on points of interest. A color mapping can be applied to overlay chronological information (Woodring and Shen, 2006). With this method, the temporal evolution is visualized by merging the spatio-temporal data into a volume. Although, there is a compromise to make between occlusion and the amount of displayed data, it is less suited for dense data. Alternatively, in order to reduce the data to display, regions of interest can be defined either manually or automatically. For example, Lu and Shen (2008) used a dissimilarity measure to extract single time steps in the volumetric temporal data, eventually displaying only the essential time steps on a color mapped timeline.

In the context of a more recent work by Johnson et al. (2019) proposed BentoBox, a Virtual Reality data visualization interface for simulated volumetric and temporal data, which juxtaposes several instances of a volumetric dataset under different parameters, in an array layout. BentoBox disposes of a range of tools focused on the comparison of datasets and manipulation of parameters, using animations, color mapping and 3D bimanual interactions.

Finally, a versatile visualization technique which enables the compact visualization and manipulation of time-varying data, is the Space-Time Cube which is detailed in the following section.

### 2.2 Space-time cube visualization

First introduced by Hägerstrand (1970) as time-space volume and paths in a context of socio-economic study, the Space-Time Cube (*STC*) is a representation using two axes for data and a third axis for time. It is used for numerous representations of time-varying data, would it be for geometrical illustration, geographic data (Ssin et al., 2019; Leduc et al., 2019) or trajectories (Filho et al., 2019). A direct usage of the STC for biological imaging, can be found in the SeeVis visualization tool Hattab and Nattkemper (2018), in which pre-processed 2D images obtained from time-lapse microscopy data are stacked into a STC for further analysis.

Bach et al. (2014) summarized, in their review of temporal visualizations based on the STC, different types of operations that can be applied to visualize data in a STC. Cutting operations consist in extracting a slice of the cube. A cut perpendicular to the temporal axis extracts an image at a particular moment in time, showing the state of an observed entity at a specific time; A cut along the time axis corresponds to the evolution of a line over time. On contrary, flattening operations collapse the STC along an axis to obtain a 2D representation; When the time axis is collapsed, it corresponds to a projection of the movement of the observed entity, on a plane, during the whole temporal window. In addition, depending on the data and the visualization design, 3D rendering, interpolation, volume extraction, non-orthogonal or non-planar operations can also be used. Later work by the same authors (Bach et al., 2017a) generalized the notion of STC visualization, modelling various visualization methods as associations of operations on a STC.

We identified two main research topics for extension of STC visualizations. First, high dimensional datasets, with more dimensions than could fit in a 2D temporal STC, could use a similar abstraction and be represented as high dimension volume. Adapted operations on this volume would need to be created to yield visualizable 2D or 3D images. If the idea of a higher dimension STC was evoked in Bach et al. (2014); Bach et al. (2017a) reviews, this lead has not been thoroughly explored. Among the works that are the closest to it, the method Woodring and Shen (2006) described previously already evoked a time flattening operation on a 3D temporal dataset; in this direction, Woodring et al. (2003) proposed a method to generate and render 3D slices based on an arbitrary hyperplane equation. In the latter contribution, the authors gave a few guidelines to interpret the output visualization with particular value of the hyperplane equation. However, the intuitiveness of the equation parameterization and concrete interpretation of the visualization have not been formally evaluated. Finally, another method was proposed by Krone et al. (2016) in which the focus was on the evolution of the surface of molecular membranes. In this scenario, a surface map of a molecule is computed, unwrapped and projected onto a plane, allowing comparison of multiple time points using a STC.

The second main research topic for STC visualizations is interactivity. Bach et al. (2017a) advice against direct 3D rendering of STCs, notably because of issues of occlusion, depth ambiguity and perspective distortion. Non-etheless, other works (Bach et al., 2017b) present methods using immersive technologies to help handle these difficulties. Such method would allow to take advantage of the general overview of the data available with direct 3D rendering. STC visualization can also take advantage to direct interactions in immersive environments. For example, the GeoGate system by Ssin et al. (2019) displays geographic trajectories (2D + time) in an augmented reality environment using a STC. A tabletop system displaying satellite information is coupled with the STC visualization to provide additional context information. Furthermore, the user can interact with the data through a tangible ring controller, providing natural interactions. In addition, Wagner Filho et al. (2019) also implemented a STC displaying trajectory datasets in immersive environment, with a basic interaction tool set. They report that it helps in usability and partially addresses the issue of the steep learning curve of the visualization method.

This literature review highlighted the lack of methods for the visualization of 3D spatio-temporal data, and especially for surface-mesh based datasets, according to Kim et al. (2017). The use of the STH, although slightly explored in the literature, showed a high promise in order to produce various meaningful views with reduced dimensionality but with the potential drawback of generating complex and cluttered visualizations. However, the benefits of Virtual Reality systems in terms of depth perception and interaction can overcome such limitations. McIntire et al. (2014) report in their review that stereoscopic displays are better than desktop displays for tasks of identification and classification of objects in cluttered static environments, overall complex spatial manipulation and spatial understanding. These are the type of tasks that can be expected during the exploration of complex static visualization, such as STCs, by analysts.

## 3 Space-Time Hypercube visualization

This section presents our main visualization method, based on the Space-Time Cube. First, we present a generalization of the STC in 4 dimensions, the STH, then we detail the design choices for the generation and visualization, as well as the interaction methods available.

As a support example for this section, we will use a dataset of a time-varying 3D spatial data, a 6-hour-long live recording of an embryo development Leggio et al. (2019). We detail more thoroughly the context of this dataset in Section 4. In this work, we use 100 of the 180 time points recorded, from a 65 to 383 cells embryo. Figure 1 shows some of the time steps that were used. The dataset is composed of surface meshes, for each cell at each time step. The objects are empty yet stacked very close together, resulting in a dense and cluttered view. The tracking of cells in time, notably after division, are also present, as well as numerical and categorical information. Movements between time points are of a lower order of magnitude than the size of the cells. The dataset will be described more thoroughly in Section 4.

### 3.1 Generalization in 4D

In a classic 3D STC, the data visualized is composed of 2 spatial dimensions as well as a temporal dimension (*x*, *y*, *t*). In contrast, a STH can contain 3 spatial dimensions and a temporal component (*x*, *y*, *z*, *t*). With this abstraction, any elementary operations applicable on a STC, notably described by Bach et al. (2014), Bach et al. (2017a), should be extendable and applicable to the STH, at the price of a higher level of abstraction. Geometry and content transformations now modify a 4D volume, filling operations now includes 4D interpolation, extraction operations can render 4D volumes and flattening operations can compress 4D data into a 3D volume. Furthermore, this complex set of operations can be exploited to design adapted complex operations for the STH, in order to create meaningful visualizations of the data. Such visualizations could represent the dimensions differently, providing new points of view and emphasizing different relations between the dimensions, especially time. Although, several constraints appear with this additional dimension. First, while a STC can be directly rendered, a STH cannot be directly rendered as is. To enable a direct visualization, a projection operation on the STH has to be defined in order to yield a 3D visualization. Second, the design of a 4D operation is not straightforward due to the difficulty to approach and visualize a 4D volume.

We designed such projection using elementary operators for STCs. Initially, we considered a flattening operation on the spatial dimension, projecting the 3D snapshots of the dataset into 2D planes, in order to reduce the dimension of the STH by one. However, microscopy imaging data is typically dense, and the data aggregation through flattening operators would be prone to occlusion issues. In contrast, a volume extraction operator would enable the reduction of the data dimension without increasing density. Thus, we opted for a volume extraction operating by defining a hyperplane laying in a 4D space. Nevertheless, two major issues remain:

• An extraction operation implies a loss of data during the creation of the visualization. Thus, the user must have control over which data is selected.
• The concept of a 4D hypercube is not easy to comprehend; the extraction of a volume along a hyperplane is even more difficult. The complexity of the model will be a constraint during the extraction operation, as well as any other interactive operation based on the 4D data.

These two constraints will influence our design choices, especially the generation process for our 3D STC visualization.

### 3.2 Extracting a 3D STC from a 4D STH

The proposed projection method relies on the use of a 4D hyperplane, manually defined by the user, to extract the 3D visualization. However, the definition of a hyperplane of this nature is an abstract task, which cannot be directly visualized. A simple example would be to define a hyperplane perpendicular to the time axis which would result on the extraction of the spatial data at a given time point. In order to ease the process and obtain a hyperplane yielding STCs containing mainly relevant information, we propose a user defined approach based on the cross-section, a commonly used tool in scientific visualization to explore spatial 3D data, extracting a 2D view from 3D data.

Precisely, the user can place an interactive clipping plane in order to get a cut-away view of 3D spatial data. With the same analogy that we used to consider our spatio-temporal data as a 4D volume, the plane, as a time-varying object in the 3D space, can be considered as a hyperplane in the 4D space. The operation thus corresponds to a projection such as (*x*, *y*, *z*, *t*) → (*x*′, *y*′, *t*), with *x*′, *y*′ the projected coordinates on the cutting plane. Such projection avoids any spatial distortion that could impair the interpretation of the output shape. Considering the whole dataset as a 4D Space-Time Hypercube, it sums up as a space cutting (see Bach et al., 2014) by a hyperplane, extracting a 3D space-time cube.

**FIGURE 2:**
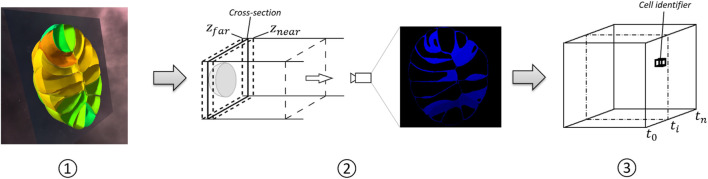
Flow diagram of the STC generation. In step 1), the user places the interactive clipping plane to get the desired cross-section. In step 2), camera parameters are automatically set in order to render the cross section at each time point. The image presents the output of the rendering operation, using the RGB channels to save cell identifiers. Stacking the rendered images yields a 3d volume, as shown in step 3). Each voxel contains a cell identifier, and its position in terms of depth indicates a time point *t*_*i*_.

The proposed generation method is data agnostic (either mesh-based or volumetric) and its three main steps are illustrated in Figure 2. 1) The user places a clipping plane on the model in order to define the “hyperplane” which will determine the projection. The clipping plane can be placed at any time point. 2) For each time step, a 2D image of the cross-section of the 3D model is computed. The rendering is achieved by setting an orthogonal camera perpendicular to the clipping plane and setting the near and far planes at a distance of *ϵ* = (*z*_*near*_ − *z*_*far*_)/2 towards the clipping plane. The field of view of the virtual camera is minimized according to the maximum size of the cross-section over time. The color channel of the rendered image encodes objects (cells in our examples) identifiers, which allows indexing numerical or categorical data during visualization. This requires object information, which implies potentially complex segmentation processing in the case of biology imaging. If this type of information is not available, raw position data can be encoded in the volume either way. 3) The output images are stacked into a 3D texture in which the RGB channel is used to encode the object identifier. The depth coordinate for each pixel encodes the time step. The use of object identifiers is considered for convenience when the spatio-temporal data has this information. In case that this information is not available, other information could be stored, such as density values from a CT or fMRI volumetric dataset.

### 3.3 Visualization: 3D rendering and interaction with numerical and categorical data

The projection operator generates a 3D texture which can be directly rendered using Direct Volume Rendering methods, as described by Stegmaier et al. (2005). To support the study of the structure, i.e., of the evolution of the cross-section selection over time, compared to additional information present in the dataset, we annotate the information through color mapping. Due to the nature of our datasets, we consider an opaque rendering avoiding semi-transparency, in order to limit color distortion when using color to encode information. We encode numerical or categorical information in textures, used as a 2-entry array, according to time and object identifier. As time and identifier are directly accessible in the 3D volume, the information to display on the visualization can be color mapped according to each object, and switched at low computational cost. We apply a Blinn-Phong illumination model on the cube to enhance depth cues and get a better understanding of the shape of the volumetric data. To reduce the rendering time, normals are pre-computed during the generation of the STC and stored in an additional 3D texture. Normals are computed using 3D Sobel-Feldman gradient operators on each direction. The result of the 3D rendered STCs, before and after color mapping, are shown in the second and third images of Figure 1.

However, the rendered STC will potentially have intricate 3D structures presenting a complex layout. The following section details manipulation techniques proposed in order to improve the exploration of the STC.

### 3.4 3D interaction in virtual reality

The complexity of the 3D structure, the occlusion and visual clutter resulting from the direct rendering led us to consider visualizing the STC in VR, in order to take advantage of the enhanced interaction and exploration capabilities offered by VR systems. Bach et al. (2014); Bach et al. (2017a) described several generic operations that could be applied to a STC, such as extraction of volumes, surfaces or curves, flattening of such extracted geometries, spatial transformations, etc. These operations can be executed dynamically, which is suited for VR interaction. Furthermore, by displaying the STC in a VR environment, additional depth cues will be available, such as binocular cues and motion parallax cues (Loomis and Knapp, 2003), which will increase the identification of the intricate structures in the STC.

In the first place, as previously mentioned, the STC is interactively generated, since the user can choose the base cross-section, and thus the hyperplane, and start the generation process at run time, doing a space-cutting operation of the 4D data. Taking advantage of the immersive environment in terms of interaction, we designed a number of interactions adapted to our data structure and the benefits it would imply for data visualization. All the techniques described in the following sections assume that the user is wearing a head mounted display (HMD) and holding by the hands two 6 degrees of freedom hand-held controllers. As such, the user will be able to explore the STC by just naturally walking in the virtual environment and use both hands to use the different tools. We refer the reader to the accompanying video which showcases the different interaction tools.

#### 3.4.1 Exploration tools

The tools described in this section are meant to help the user explore potentially dense and occluded volume representations, and also to support the execution of analytical tasks.

Clipping Plane: An interactive clipping plane generate cut-away views of the STC. The user can directly grab the clipping plane and manipulate it using direct hand translations and rotations. Yet, some orientations are easier to interpret:

• Time cutting, i.e., putting the plane perpendicularly to the Z-axis, corresponds to going through the original cross-section at given instants.
• Linear space cutting, i.e., putting the plane along the Z-axis, corresponds to the evolution of a segment of the spatial data over time, giving for instance information of the evolution of the spatial structure.
• In case of moving objects, slightly oblique cutting can help track the object over time. This type of cut was used to generate the cut in Figure 4.

Selection operator: This tool is a pointer that displays summarized information of the hovered object, such as its identifier. This is a detail-on-demand operator that can display additional information on the object on click. It appears as a red arrow object, so that the user can put the pointer precisely on the desired object, providing a highlight feedback upon hovering or selecting.

**FIGURE 3:**
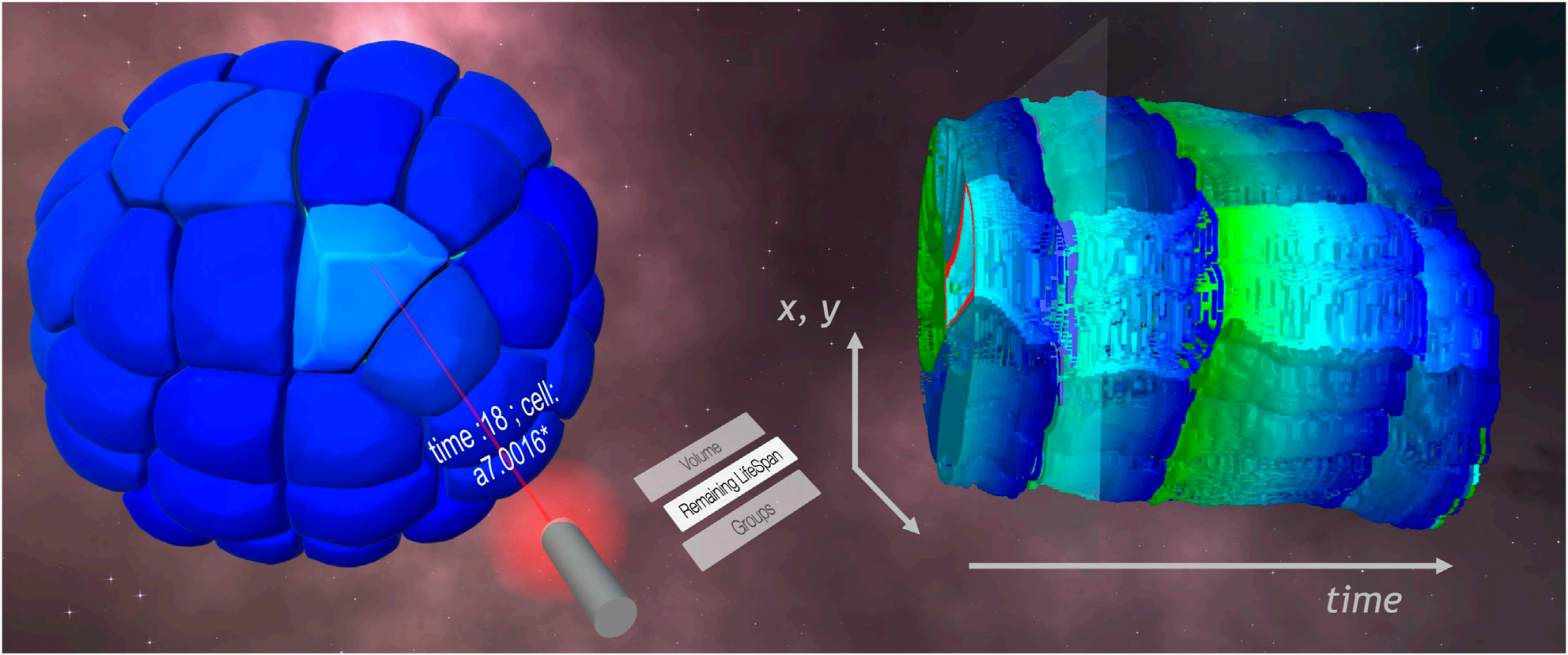
STC (right) and meshed model (left) visualizations in the VR framework. On the laser selector, a list of available information allowed the user to color map the remaining lifespan of cells on the visualizations. A cell is selected on the meshed model. The name of the cell is displayed on the pointer, and feedback appears on the STC, highlighting the lineage of the cell.

Switching the color mapped information: When various numerical or categorical information are present in the dataset, the user can scroll through the list of information displayable (cf Figure 3). As detailed in Section 3.3, the underlying operation corresponds to switching the texture containing the information, and can thus be executed interactively.

Light: The user can grab a point light source represented as a yellow sphere and manipulate it using direct hand translation. This helps understand the shape of the volumetric data by adding depth cues, reveal shape, reliefs and details of the STC.

#### 3.4.2 Filtering operations

As occlusion remains an issue in dense volumetric data, reducing the amount of data displayed becomes necessary.

Value Filter: This tool allows the user to select which objects are displayed, according to a range of the color mapped information. This method allows to identify groups of objects sharing similar characteristics and remove objects that might occlude the view. The user controls the range using a virtual slider.

Time Filter: This tool allows the user define a temporal window to constraint the data rendered in the STC within two time steps, selected using a slider. This tool is conceived to allow the user to pinpoint short temporal vents in the specified temporal window.

Object Filter and Tracking: The user can highlight or hide objects through direct selection. This feature helps the user to track the objects in time, or to find objects of interest in a cluttered environment. The user, by selecting objects using the selection operator described in Section 3.4.1, can change the state of the object—From normal to highlighted to masked—Upon clicking the trigger of the controller. The state is also propagated over time, in order to highlight all the instances of the given object over time. Furthermore, if the object follows a tree hierarchy in time, such as the cells with their children cells in our example dataset, the operation is also propagated to the children objects as well. Yet, it should be noted that to achieve such propagation, the dataset requires identifiable objects over time and hierarchy information. Figure 3 shows an example of object tracking.

#### 3.4.3 Linking multiple visualizations

We took advantage of the large workspace offered by virtual environments to display the meshed model in addition to the STC. We followed advice from Munzner (2014) for the design choices implied by this juxtaposition: multiple views should be coordinated, and even interactively coordinated, so that elements from one visualization could be contextualized in the other, despite different information or level of abstraction. Consequently, the operations we described above are applied to the STH, and feedback of these operations is given through the two visualizations displayed—The STC and the meshed representation. Links can then be set up between the visualization by applying a shared encoding of the effect of the operations.

Linking the time exploration: In addition to the time filtering sliders evoked previously, a middle cursor controls the current time displayed on the meshed model. Feedback of this cursor appears as a transparent plane orthogonal to the temporal axis of the STC, as shown in Figure 3.

Linking the spatial exploration tools: The same color mapping is applied to the STC and meshed model, notably after switching the color mapped information interactively, as shown in Figure 3. The clipping plane used on the meshed model to generate the STC can go back on click to a default position, which is the one of the displayed STC’s base cross-section. A marker line on this cross-section gives context information about the position of the clipping plane applied to the STC. This line corresponds to the intersection between the STC’s clipping plane and the temporal feedback plane.

Linking the filters: Any filtering operation done on the STC is also applied to the meshed model, and *vice versa*. The same objects are highlighted, hidden or filtered out by time or value.

These operations were designed to help users to explore temporal evolution with the static visualization offered by the STC, and correlate the multidimensional data or pinpoint events by combining the two visualizations and the interactive tools available, as illustrated with the following use case.

### 3.5 Performance: High frame rate navigation

The VR application runs on Unity 2018.2.21 and is supported by a PC with Windows 10, an Intel Xeon W-2104 CPU (4 cores, 3.2 Ghz base frequency) and a RTX 2080 GPU. All interaction methods described previously are designed in order to maintain a framerate above 45 fps.

In terms of performance and texture resolution, the STC used in the figures has a resolution of 256 × 256 × 100. The full generation process takes about 5 s. This process is GPU-friendly, which enables the generation of the STC during runtime. Normals are for instance generated and saved instantly using compute shaders. However, the bottleneck of the current implementation of the generation algorithm is the CPU - GPU transfer, provoking a framerate drop.

## 4 Use case: Application to cell division and morphogenesis analysis

In this section, we demonstrate the potential of the STH applied to the visualization and analysis of spatio-temporal data from the recordings of embryonic developments of a *Phallusia mammillata*, a marine invertebrate. We present the studied dataset, which has been already shown in the previous section, and detail a VR application in which the STH has been integrated. Finally, we describe three use cases, based on recommendations for classic analytical tasks by the domain experts we worked with, illustrating the potential benefits of the STH.

### 4.1 Embryonic development dataset

The data set used to illustrate the STH data is a live recording of the embryonic development of the *Phallusia mammillata*, a marine invertebrate animal of the ascidian class. The embryo was imaged every 2 minutes for 6 hours using confocal multiview light-sheet microscopy, generating a 4D dataset with isotropic spatial resolution of several terabytes (Guignard et al., 2017). The data was then segmented using the ASTEC pipeline (Adaptive Segmentation and Tracking of Embryonic Cells) designed by Guignard et al. (2017), and meshed using the VTK library (Schroeder et al., 2004) and MeshLab (Cignoni et al., 2008), producing a surface-based spatio-temporal dataset of a few hundred megabytes representing the embryo. Examples of the surface meshed model of some of the 180 time points recorded are shown in Figure 1.

In addition, the ASTEC pipeline extracted global lineage trees of the embryo, i.e., the tracing of cellular genealogy derived from cell divisions and migration. Other categorical or continuous data, such as the volume of cells or the remaining lifespan—i. e., time before the next division—Were computed or added by the community of experts working on the dataset. We also had access to the ANISEED (Brozovic et al., 2017) database, providing us with categorical information about the expression of various genes in the cells.

### 4.2 VR visualization application

Taking advantage of the large workspace offered by immersive environments, we developed a VR application for the user to interact with the meshed model, in addition to the STC visualization. We based our environment on the framework *MorphoNet*, an online interactive browser for the exploration of morphological data, by Leggio et al. (2019). The development of the application was done using Unity 3D and the HTC Vive was the main visualization and interaction system.

Our framework displays the dataset described above using meshed model of the embryo at each time point, as well as a STC based on a cross-section placed close to the median plan of the embryo, as shown in Figure 5. The color mapped on the visualizations here corresponds to the volume of the cells.

Regarding the user interface, a virtual desk contains all the available tools that the user could grab using the trigger button of the HTC Vive controller. Once a tool is grabbed, it can be used either using the trigger, or by performing circular motions on the circular touch pad of the HTC Vive controller. This last interaction allows to control a continuous value (e.g., the time) or to scroll on a list. The user could grab a tool in each hand.

The different designed interactions were a laser selector to select a cell (see Figure 3), a screen to display information on the selected cell and a remote control clipping plane, also used to select the plan for the STC generation. In addition, a bi-manual hand manipulation, similar to a remote Handlebar technique (Song et al., 2012), allows applying rotations to the meshed model by holding of the trigger button on both controllers. This can be enabled at any time, even while using a tool. The meshed model is virtually attached to an axis defined by the position of both hands. In order to activate the rotation, users have to press the trigger of both controllers. The modification of the length of the user’s hands gap modifies the scale of the meshed model. The rotation can also be applied to the STC, depending on which visualization the users is looking at.

This set of tools, as well as the ones dedicated to the STC, provides the user with primary controls to explore the dataset.

### 4.3 Use cases: Efficient visualization of divisions or deformation

To study functional organization and arrangement of the tissue, embryo developmental studies remain essential to access cellular functions inside a self-organized isolated system. With a fast development, a few hundreds of cells, ascidian embryo, like the *Phallusia mammillata*, is the perfect choice to analyze the link between cells or tissue geometries and differentiation. In the particular case of the *Phallusia mammillata*, the cell membranes are very transparent, which makes for an easier imaging of the inside of cells through light sheet microscopy. Without any apoptosis, i.e., programmed cell death, or cell migration in early ascidian development, embryo topological complexity can be summarized in the result of unequal divisions of cells, resulting in two different volumes for each daughter cell, and/or asynchronous divisions, i.e., two daughter cells having different cell cycle duration. Each of these events correlates with cell fate decision of the cells. This way, with the exploration of cell architecture and adjacency in a dynamical view in embryos, we have access on a major part of the story and decisions taken by each cell.

We will illustrate in the following examples of use of the STC to observe events and behaviors with a strong temporal component related to the exploration issues described above. For this purpose, we generated a STC from the embryo cross-section shown in Figure 5.

#### 4.3.1 Cellular deformation

One of the main steps of the embryonic development for most animals is gastrulation. It is characterized by morphogenetic movements that form a cavity in the embryo. These movements set the base of the determination of the morphology of the future individual. The actual source of these movements and deformations of the cells is still unsure, and usual measurements of deformation can be insufficient to describe such movement thoroughly. Therefore, visualization of this deformation over time is necessary for the analysis of the embryonic development.

**FIGURE 4:**
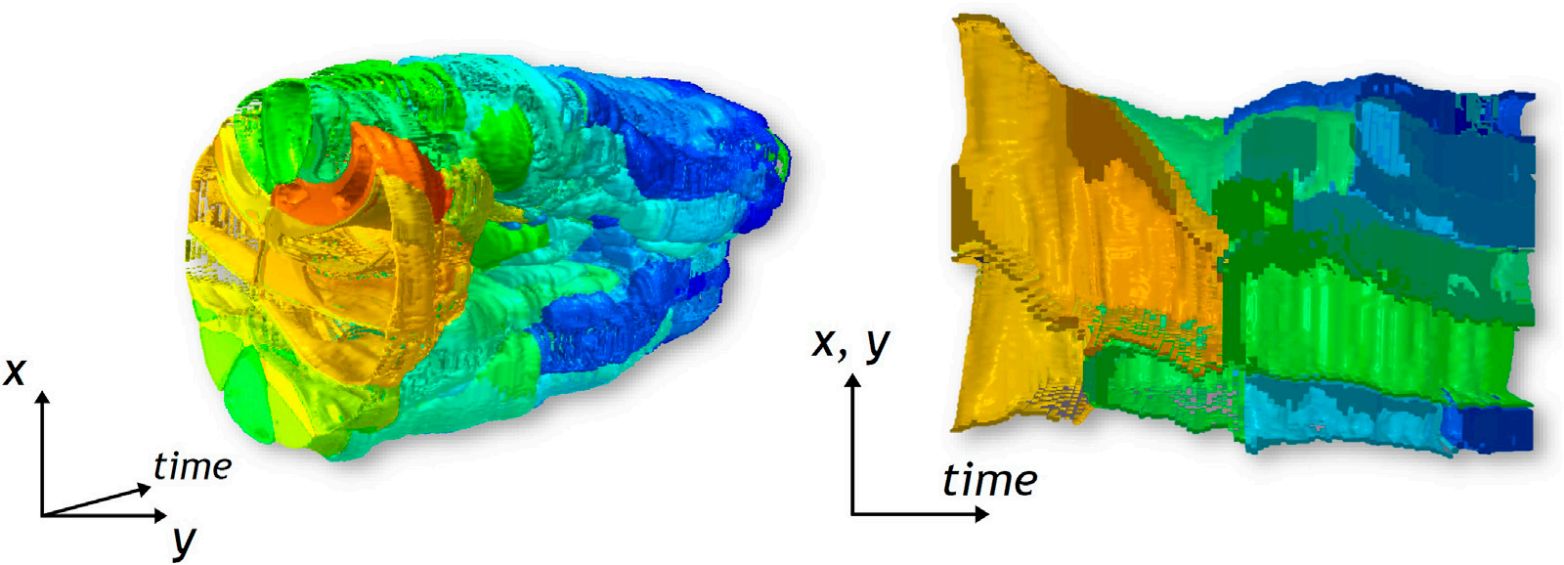
The STC at the left presents the cavity formed by gastrulation. The cross-section at the right shows the deformation of cells involved in this particular morphogenetic movement.

The STC shows the deformation of the embryo over time, as shown in Figure 4. A cross-section allows the exploration of the main cells involved in the movements and provides a view of the global and local deformation. The user can then identify the cell to visualize by selecting them on the STC and continue the exploration on the 3D representation.

#### 4.3.2 Waves of cell divisions

During the early stages of the embryonic development, cells tend to divide approximately simultaneously. This phenomenon results in “waves” of division, during which numerous cells divide within a short time.

By sliding through the time component, one can notice this kind of event on the surface-based representation through several aspects. First, the characteristic dynamics of the membranes of a cell can be observed on a large number of cells: on the surface of the embryo, and inside by using a clipping plane. Second, as cells divide, they reduce their volume. Thus, the color mapped on the embryo will change a lot during such event.

**FIGURE 5:**
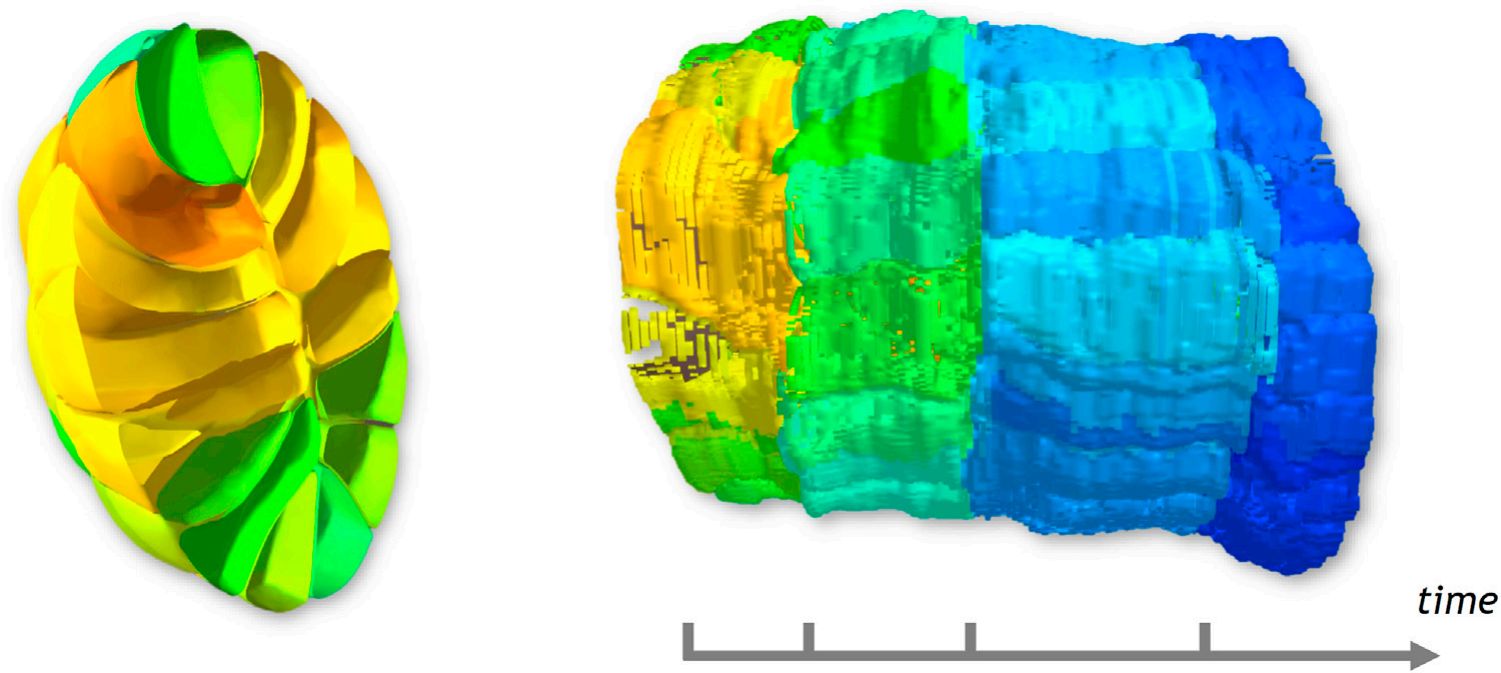
Cross-section and related STC generated. The color displayed on the STC corresponds to the volume of cells, and helps distinguish 4 parts.

Keeping this last property in mind while exploring the STC, color clusters become distinguishable thanks to the volume information displayed. This observation can thus be done directly, without having to do a time-sliding operation, as shown in Figure 5. Up to four color clusters, hence three waves of division, can be identified.

#### 4.3.3 Asynchronous behavior in cell divisions

Regarding cell determination, cells with different fates will not result in the same number of cells, and embryonic development has to be controlled. Thus, at specific moments, some cells will not divide at the same time as the other ones and skip a wave of division.

Exploring the surface-based representation in order to find cells presenting this behavior, the user has to slide back and forth in the time window corresponding to the wave of division and observe which cell will not divide.

**FIGURE 6:**
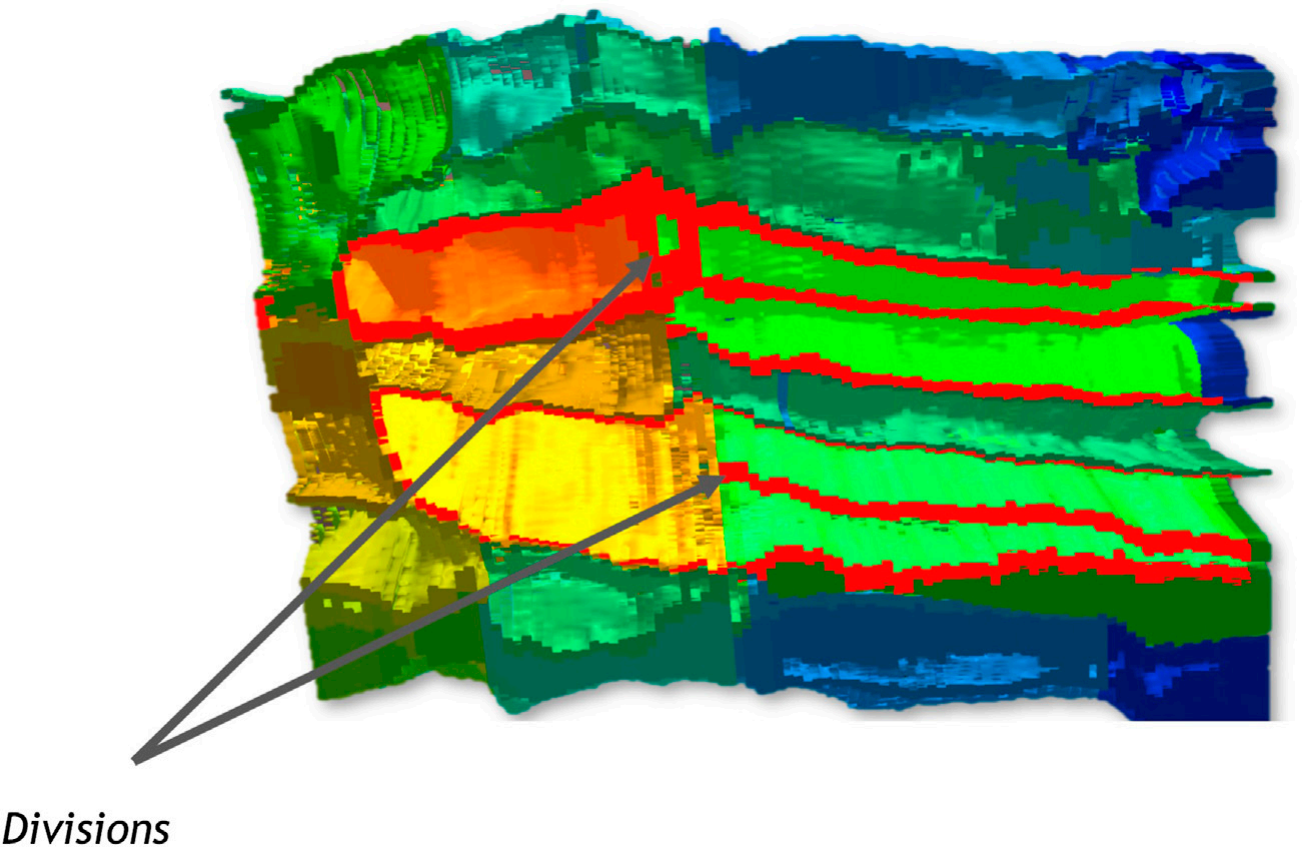
Cross-section of the STC. Two divisions are indicated. The membrane of the original cells and their respective daughters cells are highlighted in red.

The STC can give a more global view of this time window, with a part of the context of the embryo. In Figure 6, membranes of a cell are highlighted in red. Only one division can be observed for each of these cells during the time window represented. Up to three were to be expected here, according to the waves of division identified before, which confirms an asynchronous behaviour for these cells. With the feedback of the cell selection tools on the STC, the user can find quickly the related cell on the 3D model of the embryo.

### 4.4 Expert feedback analysis

During the design and development of our method, five domain experts helped us by providing advice to formalize the use cases presented before to test the method, as well as the priorities of biologists in terms of tool to approach such use cases, and by testing the application. In addition to informal discussions during the design of the system, the domain experts tested the application in two different sessions, at different stages of the development of the prototype. They all participated to both sessions, which were 3 months apart, for 1 hour for each expert and session. The domain experts had no previous experience with Virtual Reality, three of them had experience with the *MorphoNet* framework and the dataset. The two others had no experience with either.

The first session consisted in a presentation of an early version of the visualization method, that did not include most interactions of filtering and selection. We presented the concept of the STH and the generated STC to the biologists, as well as the benefits of immersive interfaces for the analysis of their datasets or our visualization method. Then, they were asked to try the VR application freely, interacting with the available tools, the classic mesh representation and the STC. The focus of this session was to test the overall usability of the system, their first impressions on the STC visualization, and potential integration of VR applications in their usual workflow. At first, the participants were skeptical, both about the visualization, as it was initially presented on 2D images; and about the VR interface, which some of them considered unnecessary. This impression changed during the tests, such that one of the users declared how they needed “to be actually seeing the STC in 3D to really understand what was going on”. At the end of the session, all of them estimated that they were able to interpret the visualization and to use it to identify temporal behaviours in the data. Non-etheless, they had some difficulty adapting to the interface. Each analytical tool was handled pretty quickly and was deemed rather user-friendly, yet the amount of tools available, even in this preliminary version, created a important workload. The users visibly struggled finding the right tool and designing fluid strategies to complete simple tasks. We recorded their difficulties and enhanced the application before the second session.

In the second session, the domain experts had access to the whole analytical tool set, and were again asked to use the STC freely. Although, this time, they were also given the objective to use the visualizations to identify the specific events described in Section 4.3. These tasks were proposed as exercises to introduce the STC as an Immersive Analytics tool, even if it did not completely corroborate with usual tasks achieved by the analysts. During this session, the users required less time to accommodate to the VR interface and the application: they overall reported that the application easier to use than the previous time, both because of their experience and because of the improvements. Regarding the execution of the analytical tasks, the participants were unanimous on its utility for the wave detection task. Non-etheless, opinions were mitigated on the asynchrony and division detection tasks: we noticed that some of them struggled a bit more depending on their choice of tools, ease to use each of them effectively and strategy of analytical workflow. In other remarks, the STC was deemed “similar to a cell lineage tree (Sulston et al., 1983) but with spatial and especially neighbourhood information”; The latter giving a supplementary dimension in the analysis of the data. This, aided with adequate color mapping, could be relevant in cases of analysis of signals or communications between cells for example.

Few other improvements were suggested during these tests, most of them regarding the addition of further information in the environment, or enhancing some controls. The need of comparing and linking different datasets was also especially pointed out, echoing with Kim et al. (2017) observations.

## 5 General discussion

While the evaluations described in the paper have showed the potential usages and advantages of the STH for the visualization of 3D spatio-temporal morphogenesis data, there is yet a number of additional future works in order to cope with its current limitations. First, the generated STC visualizations are based on a projection operator which enables the visualization of a subset of the original 4D data. Although this approach generates a compact representation of the spatio-temporal data, it also results with a loss of spatial information and can be sensible to strong motions within the dataset. For example, strong motions can be particularly problematic for objects close to the cutting plane that can result in wrong interpretations (e.g., an object shrinking when it is just moving away from the clipping plane). In the current implementation of the system, we tried to compensate for these negative effects by the juxtaposition of the meshed model, which gave additional contextual information missing in the STC visualizations. Nevertheless, future works should explore how to adapt the projection operation to extract more reliable information. For example, the clipping plane could be adjusted over time to track an object of interest or consider the internal motions of the objects, potentially extending the cutting plane to an arbitrary surface.

**FIGURE 7:**
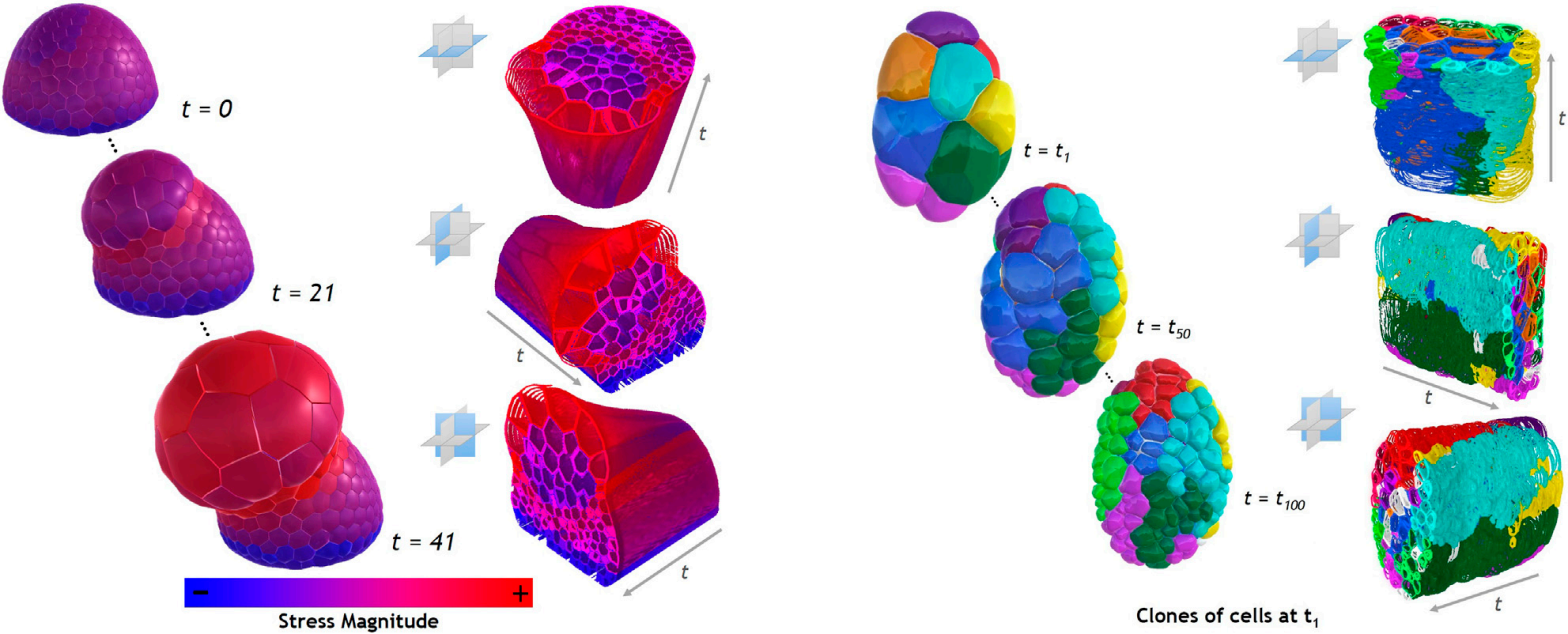
Examples of STCs based on 3 different base planes for the capture, on each example dataset presented in Section 5. The left side of the image is extracted from the shoot-apical meristem dataset, colormapped with numerical values of stress magnitude. The images on the right side are extracted from the *C. elegans* embryo dataset. Each color represents the successive divisions of the cells present at *t* = *t*_1_.

A second aspect that should be further studied, is the usage of the STH for other spatio-temporal datasets. In order to complement the examples shown in this paper, we provide additional generated STCs from two additional datasets. The first dataset shown in Figure 7, left side, is a simulation in 42 time points of an abstract oryzalin-treated organ. The appearing outgrowth is obtained by locally softening the membrane of a shoot-apical meristem filled with uniform and steady turgor pressure. The stress magnitude on each cell, notably implied by this outgrowth, is colormapped on the STCs. The second dataset, shown in Figure 7, right side, is part of a 3D temporal imaging atlas of cell morphology for the *C. elegans* embryo. The recording includes 150 time points, from the 4 to 350 cells stages. The STCs displayed show the remaining lifespan of each cell as a colormap1. However, the examples and use cases described in this paper are related to the analysis of morphogenesis. In these datasets, the temporal and spatial coherency was high, i.e., motions between two consecutive data points were relatively low, with quite a low spatial density, and the different structures visualized are well-defined (i.e., cells). The STC visualizations had a number of cavities which allowed a good tracking of the cells and were easily explored using the additional cutting operations. It remains unknown whether the STH approach would be still usable for spatio-temporal datasets with lower temporal and spatial coherency, and higher spatial density. In such situations, the STC visualization could be denser and less continuous, potentially impacting its comprehension. Nevertheless, the usage of the STH will still be dependent of the purpose of the analyst.

A third aspect that should be also noted is the exploration of other direct volume rendering methods. Indeed, a wide range of methods existing for the enhancement of DVR rendering methods (Jönsson et al., 2014), particularly with the objective of improving the comprehension of the complex and interacted structures that volumetric datasets have (Preim et al., 2016). In the presented work, we leveraged stereoscopic viewing for improve the understanding of STC rendering, but other use cases might require the highlight of certain structures (Bruckner and Gröller, 2007) or to consider other shading methods (Díaz et al., 2017). Further work could explore the role of additional rendering methods, and in particular the use of transfer functions with different levels of opacity of the understanding and interpretation of the generated visualizations.

Finally, a recurrent comment from the domain experts was the addition of annotation functionalities to the system. In this respect, in the morphogenesis context, domain experts considered that the tool could be of interest for data curation, in particular to validate/complete the object tracking algorithms over time, notably after cell divisions, or to label cells. Such operations can be very cumbersome on desktop applications due to the multidimensional nature of the data. The STC visualizations could help for detecting errors or areas of interests and use them to directly annotate those events.

## 6 Conclusion

In this paper, we have proposed a novel spatio-temporal visualization based on the Space-Time Cube visualization. The proposed visualization, the Space-Time Hypercube, extends the STC visualization to consider a third spatial dimension in the data. To enable a direct visualization, we proposed a projection operator on based on a user-driven cross-section defined in 3D space. The projection of the hypercube, consequently a STC, contains only a partial spatial information of the dataset, but creates a view containing temporal information. Numerical and categorical information could be displayed as well on the visualization. We juxtaposed and linked the STC and original dataset visualizations in a VR application, taking advantage of immersive environments benefits in terms of visualization and interaction in 3D. Various tools for exploration, filtering or tracking objects apply transformations on both visualization and come to assist in the analysis of the dataset. Moreover, we illustrated the potential usages of the STC in the context of morphogenesis. The evaluation with domain experts showed that the STC visualization presents a number of benefits with respect to traditional visualizations, especially for the detection of events with a temporal relevance. The STH approach could pave the way to new types of visualization and interaction methods for 3D spatio-temporal data, and we believe that such tools will help the adoption of VR technologies for data visualization.

## Data Availability

Publicly available datasets were analyzed in this study. This data can be found here: https://morphonet.org/dataset under “Phallusia mammillata embryo (Wild type, live SPIM imaging, stages 8-17)”.
